# Evaluation of Morpho-Physiological and Yield-Associated Traits of Rice (*Oryza sativa* L.) Landraces Combined with Marker-Assisted Selection under High-Temperature Stress and Elevated Atmospheric CO_2_ Levels

**DOI:** 10.3390/plants12203655

**Published:** 2023-10-23

**Authors:** Merentoshi Mollier, Rajib Roychowdhury, Lanunola Tzudir, Radheshyam Sharma, Ujjal Barua, Naseema Rahman, Sikandar Pal, Bhabesh Gogoi, Prakash Kalita, Devendra Jain, Ranjan Das

**Affiliations:** 1 Department of Crop Physiology, College of Agriculture, Assam Agricultural University, Jorhat 785013, Assam, India; 2 Department of Genetics and Plant Breeding, School of Agricultural Sciences, Nagaland University, Medziphema 797106, Nagaland, India; 3 Department of Plant Pathology and Weed Research, Institute of Plant Protection, Agricultural Research Organization (ARO)—Volcani Institute, Rishon Lezion 7505101, Israel; 4 Department of Agronomy, School of Agricultural Sciences, Nagaland University, Medziphema 797106, Nagaland, India; 5 Biotechnology Centre, Jawaharlal Nehru Krishi Vishwa Vidyalaya, Jabalpur 482004, Madhya Pradesh, India; 6 Plant Physiology Laboratory, Department of Botany, University of Jammu, Jammu 180006, Jammu and Kashmir, India; 7 Department of Soil Sciences, Assam Agricultural University, Jorhat 785013, Assam, India; 8 Department of Molecular Biology & Biotechnology, Rajasthan College of Agriculture, Affiliated to Maharana Pratap University of Agriculture and Technology (MPUAT), Udaipur 313001, Rajasthan, India

**Keywords:** climate change, elevated CO_2_, grain yield, high temperature, North-East India, rice landrace, SCoT marker

## Abstract

Rice (*Oryza sativa* L.) is an important cereal crop worldwide due to its long domestication history. North-Eastern India (NEI) is one of the origins of *indica* rice and contains various native landraces that can withstand climatic changes. The present study compared NEI rice landraces to a check variety for phenological, morpho-physiological, and yield-associated traits under high temperatures (HTs) and elevated CO_2_ (eCO_2_) levels using molecular markers. The first experiment tested 75 rice landraces for HT tolerance. Seven better-performing landraces and the check variety (N22) were evaluated for the above traits in bioreactors for two years (2019 and 2020) under control (T1) and two stress treatments [mild stress or T2 (eCO_2_ 550 ppm + 4 °C more than ambient temperature) and severe stress or T3 (eCO_2_ 750 ppm + 6 °C more than ambient temperature)]. The findings showed that moderate stress (T2) improved plant height (PH), leaf number (LN), leaf area (LA), spikelets panicle^−1^ (S/P), thousand-grain weight (TGW), harvest index (HI), and grain production. HT and eCO_2_ in T3 significantly decreased all genotypes’ metrics, including grain yield (GY). Pollen traits are strongly and positively associated with spikelet fertility at maturity and GY under stress conditions. Shoot biomass positively affected yield-associated traits including S/P, TGW, HI, and GY. This study recorded an average reduction of 8.09% GY across two seasons in response to the conditions simulated in T3. Overall, two landraces—Kohima special and Lisem—were found to be more responsive compared to other the landraces as well as N22 under stress conditions, with a higher yield and biomass increment. SCoT-marker-assisted genotyping amplified 77 alleles, 55 of which were polymorphic, with polymorphism information content (PIC) values from 0.22 to 0.67. The study reveals genetic variation among the rice lines and supports Kohima Special and Lisem’s close relationship. These two better-performing rice landraces are useful pre-breeding resources for future rice-breeding programs to increase stress tolerance, especially to HT and high eCO_2_ levels under changing climatic situations.

## 1. Introduction

Climate change has significant implications for agriculture and food production [1]. The changes in temperature, precipitation patterns, and extreme weather events associated with climate change can impact various aspects of agricultural systems, including phenology, growth and development, crop yields, and overall food security [2,3]. High temperature and elevated carbon dioxide (eCO_2_) levels are two significant consequences of climate change that can have profound impacts on rice-based (*Oryza sativa* L.) agriculture [4,5], which is one of the most important cereals, serving as the staple food for approximately 67% of the world population [6]. Heatwaves can cause stress to rice plantations by exceeding their temperature tolerance thresholds, leading to reduced photosynthesis, impaired pollen development, and lower crop yields [4]. Higher CO_2_ concentrations can initially stimulate rice growth due to increased photosynthesis rates, which can lead to potentially higher yields [7]. However, it is essential to carefully consider the possible advantages of eCO_2_ in light of the adverse consequences associated with rising temperatures [8]. Based on the current trends, global temperatures can be expected to rise by 3 °C at the end of the century, which will lead to fluctuations in climate-related parameters, directly and indirectly impacting on agricultural rice production [9].

Rice is the world’s most important cereal crop followed by wheat and provides approximately 50% of calories for almost half of the world’s population and its demand will increase by approximately 28% by 2050 [10]. With no CO_2_ interplay, global rice yield could decline by an average of 3.2% for a 1 °C increase in global ambient temperature. This amount could be uplifted in some regions due to increased temperatures, changing rainfall patterns, and abnormal weather events [11]. Rice is predominantly cultivated and consumed in Asia—especially in India, China and Japan, where India largely produces *indica* rice [12]. It possesses exceptional genetic diversity, making it one of the few species with such a rich genetic pool on the Indian subcontinent. Through its wide range of domestication history, rice landraces representing traditional and locally adapted varieties, hold immense potential in addressing challenges posed by climate change [13,14]. The North-East (NE) region of India is recognized as a significant hotspot for rice genetic resources due to its diverse rice-growing environments and as a secondary the center of origin for rice; the NEI possesses a rich biodiversity of rice germplasm that showcases unique characteristics [15]. As a result of the Green Revolution, most of the ancient rice varieties and landraces are still not cultivated in the majority of the rice-growing areas as they have been replaced by high-yielding modern cultivars [14]. Being the center of rice domestication, the tribal and rural areas of NEI harbor a remarkable rice genetic diversity maintained by farmers and exhibit variations in various traits, such as phenology, plant height, photoperiod sensitivity, grain size and shape, aroma, cooking quality, and tolerance to abiotic and biotic stresses [16]. Harnessing the genetic diversity present in these landraces can contribute to the development of new rice varieties that can thrive under changing climate conditions. By incorporating traits like early flowering and maturity, higher pollen vitality and reproductive success, higher biomass, improved plant traits for breeding purposes, and improved grain filling and yield, these landraces offer a promising avenue for sustainable rice production under escalating climate challenges, ultimately bolstering food security for both local communities and the broader population [17,18,19].

In addition to the breeding values of these landraces under high temperatures and eCO_2_ levels, it is also very important to the genetic characterization of the landrace population by using molecular genetic markers and to support genotypic performance to screen out better-performing landraces for future breeding pipelines. Different molecular markers, like Start Codon Targeted (SCoT), simple sequence repeat (SSR), and single nucleotide polymorphic (SNP) markers, have been used to characterize rice genotypes and have revealed their profound role in varietal discrimination and trait selection [20,21]. SCoT markers are a type of molecular marker used in crop genetic analysis and are based on the polymorphism on short ATG start codon region of genes, making them valuable tools for studying genetic diversity, population structure, and phylogenetic relationships among different rice varieties with stress-tolerance properties [22]. SCoT-marker-based analysis presents distinct advantages over other widely used molecular markers, like SSR and SNP, in rice as it does not require prior knowledge of genomic sequences for primer design, making them particularly valuable for non-model species or less-studied rice varieties [23]. Furthermore, SCoT markers offer higher polymorphism rates compared to SSRs, enabling more accurate discrimination between closely related genotypes and enhancing the resolution in genetic diversity studies. In comparison to SNP markers, SCoT analyses are cost-effective, as it avoids the resource-intensive processes of SNP discovery and genotyping. SCoT markers also target non-coding regions near start codons, potentially revealing regulatory elements that influence gene expression and functional variations. These features collectively render SCoT-marker-based analyses an efficient and versatile tool for studying trait-associated variations in rice, particularly in scenarios where genomic resources are limited or when budget constraints are considered [24].

Considering NEI’s (the Nagaland region of India) geographical location within the Indo-Burma region is considered the center of the origin of rice, this study hypothesizes that the rice landraces native to Nagaland possess unique characteristics and genetic traits developed through the evolution in this region. Due to their collection through participatory rural appraisal approaches, certain rice landraces have been identified as showing notable environmental stress tolerance, and it is anticipated that these landraces, which evolved in the center of the origin of rice, might exhibit a wide array of adaptive mechanisms in response to changing climatic parameters, including high temperature and eCO_2_. In this regard, this study aimed to identify and understand the diverse responses of Nagaland’s rice genotypes to these changing environmental factors, providing critical insights into their adaptation mechanisms. Hence, the present investigation aims to (a) characterize the native North-Eastern *indica* rice landraces for phenology, morphological, physiological traits, and yield-associated parameters; (b) study the effect of high temperature and eCO_2_ on the rice traits for improved yield; and (c) the marker-assisted selection of potential rice landraces that are better adapted to the aforesaid stress conditions for the identification of resilient traits to develop climate-resilient rice varieties.

## 2. Materials and Methods

### 2.1. Plant Materials

A total of 75 locally grown rice landraces were collected from different rice-growing places of Nagaland (Table S1) situated in North-East Indian agro-ecological regions, and were agronomically evaluated in the experimental field (26°45′ N latitude, 94°12′ E longitude, 87 m altitude above mean sea level) of Assam Agricultural University (AAU), Jorhat, Assam, India, during two consecutive rice growing seasons of the year 2019 (year-1) and 2020 (year-2). The meteorological data during the cropping seasons (from late June to late December) were obtained from the Department of Agrometeorology, AAU, Jorhat, India, and presented graphically in Figure S1.

### 2.2. Screening the Genotypes for High-Temperature (Heat) Stress Tolerance

All 75 rice landraces were screened for high-temperature (heat) tolerance (HT) by the in-field standard Screening Evaluation Score (SES) system of the International Rice Research Institute (IRRI), Philippines [25]. A completely Randomized Design (CRD) with two replications was followed for the field evaluation of seven heat-tolerant genotypes, viz. Tatza, Kohima Special, Kakadhan, Laldhan, Tzumma, Lisem, and Mapok Temeseng, which were selected for further detailed evaluation (Table 1). For comparison, one national rice check variety Nagina 22 or N22 was included.

### 2.3. Experimental Set-Up and Treatments of High-Temperature and eCO_2_ Stress

**Figure 1:**
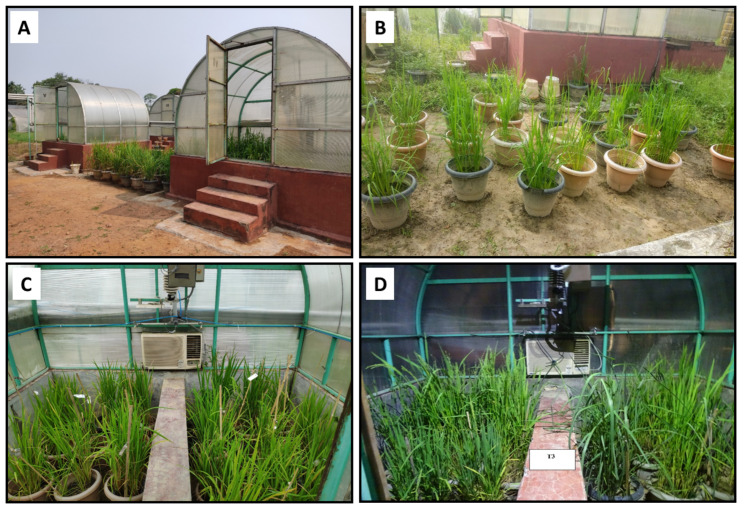
Representation of experimental setup in this study. (**A**) Bioreactor where the rice plants were grown in pots, (**B**) control condition (T1), (**C**) treatment 2 (T2), and (**D**) treatment 3 (T3).

Surface-sterilized (with 0.1% sodium hypochlorite) rice seeds of the selected eight genotypes were sown in plastic cups containing rice field soil with organic matter (50:50) and grown for 30 days. Then, 10 healthy plantlets were transferred to a bigger pot (30 cm^3^) in five replications with the same soil mixture for each treatment and maintained in the bioreactors (Genesis Technologies, Bhosari, Maharashtra, India) established in the Stress Physiology Laboratory, Department of Crop Physiology (AAU, Jorhat, India). Such bioreactors were used for further study with high-temperature and eCO_2_ stress treatments. A Factorial Completely Randomized Design (FCRD) with two factors (two bioreactors per treatment) and 5 replicates was employed for the stress treatments. Treatment-1 (T1) is the control set and comprises the maintenance of the plant pots under ambient temperature and CO_2_, whereas treatment-2 (T2) comprises CO_2_ (550 ± 20 ppm) with + temperature (ambient + 4 °C), and treatment-3 (T3) comprises CO_2_ (750 ± 20 ppm) with + temperature (ambient + 6 °C). Each treatment was maintained in individual bioreactors as follows (Figure 1):

T1: Control (ambient CO_2_ and temperature);

T2 (mild stress): CO_2_ (550 ± 20 ppm) with + temperature (ambient + 4 °C);

T3 (severe stress): CO_2_ (750 ± 20 ppm) with + temperature (ambient + 6 °C);

### 2.4. Growth Conditions in Bioreactors

Inside the bioreactor, the CO_2_ level was maintained throughout the rice plant growth during 09:00–14:00 daily. The elevation of temperature was maintained from the tillering stage to just before maturity through an infrared (IR) heater regulated by SCADA software v 5.1.6. Temperature transmitters based on Resistance Temperature Detectors (RTDs) sensors were placed in each chamber to obtain data in the control room through four core-shielded cables. IR heaters are fitted inside the bioreactors to elevate the temperature and air conditioners are placed inside, which are operated with the help of a remote controller. All the data were recorded in the computer placed in the control room.

### 2.5. Phenotyping of Phenological, Morphological, Physiological, and Yield-Associated Traits

All the agronomic practices were followed during the growth condition, as per the desired standard. Phenological parameters, like days to flowering (DtoF) and days to physiological maturity (DtoPM), were recorded at particular growth stages, as per the BBCH scale [26]. The former represents the number of days taken from sowing to the initiation of 50% flowering of panicles in a pot. The physiological maturity is expressed as the number of days taken from sowing to 70–80% crop maturity (late grain-filling stage). The canopy temperature (CT, °C) can help to optimize irrigation, detect water availability and the crop’s overall physiological condition, and is measured by an infrared thermometer (IRT) within six hours after treatment. Agro-morphological parameters, such as the s number of tillers plant^−1^ (TL/P), leaf area (LA) (cm^2^), leaf area index (LAI), relative leaf water content (RLWC), root length (RL, cm) and volume (RV, cm^3^), root biomass (RB, g), anther length (AnL), and pollens anther^−1^ (P/A), were recorded during the grain-filling stage. The number of tillers per hill (main plant) was counted on the tagged plants in the pot. The numbers of green leaves (LN) and LA were measured using a portable leaf-area meter (LI-3000C, Licor, Bad Homburg, Germany) by scanning the leaf from petiole to the tip portion, rolling out through the machine and measuring length, width, and area. LAI expresses the ratio of leaf surface to the ground area occupied by the plant as per Evans [27]. LA and LAI are commonly used as an indicator of canopies’ capability to intercept light and perform photosynthesis. RLWC can assist in the timely implementation of water requirements, plants’ response to stress, and irrigation strategies to ensure optimal growth and yield. It was calculated as per the formula used by Barrs and Weatherley [28] using twenty-leaf discs. A Soil–Plant Analysis Development (SPAD) chlorophyll meter (SPAD-502 plus, Konica Minolta, Tokyo, Japan) was used to obtain SPAD values (SPAD units) from the four uppermost fully expanded leaves on each plant during the grain-filling stage as per Frankin et al. [29]. Such a SPAD meter is a handheld device that provides a quick, non-destructive measurement of the relative chlorophyll content in a leaf and works based on the principle that chlorophyll content is directly related to the greenness of a leaf. The normalized difference vegetation index (NDVI) was recorded at the same time using a GreenSeeker handheld sensor (NTech Industry Inc., Ukiah, CA, USA) as per Kimaro et al. [30]. The NDVI is widely used to monitor and assess vegetation health, estimate biomass production, and detect environmental stress effects. For root characteristics, a set of plants was uprooted very carefully during maturity, the roots were separated from above-ground parts, and root length (RL) was evaluated. Root volume (RV) was determined by the water displacement method used by Raja and Bishnoi [31]. Then, the roots were dried in an oven at 80 °C for 3 days and the root biomass (RB) was recorded using an electronic weight machine (Shimadzu ELB600, Kyoto, Japan). AnL (as mm) and P/A were calculated at the time of flowering after staining the anthers with safranine and observed under a light microscope [32]. Plant height (PH, cm) was measured at maturity with the help of a meter scale from the base of the plant to the apex of the main axis. Yield components, such as the number of spikes panicle^−1^ (SP/P), spikelet fertility^−1^ (SF%), the number of filled grains panicle^−1^ (G/P), thousand-grain weight (TGW, g), grain yield (GY, g plant^−1^), and harvest index (HI), were measured at maturity. During maturity, all above-ground plant parts (vegetative + reproductive) of each plant with tillers per pot were manually harvested as shoot biomass (SB), and vegetative parts were separated from the panicle. Five random panicles were collected from each pot, and the number of spikes and filled grains were counted for each panicle. The spikelet fertility was estimated as the ratio of the number of filled grains to the total number of florets. Then, the grains were dried and threshed manually. The number of grains and their weight (GY and TGW) were measured using an electronic weight machine (Shimadzu ELB600, Kyoto, Japan). HI was measured as the proportion of GY to the total above-ground dry matter per pot and expressed in percentage (%).

### 2.6. Genotyping Using Start Codon Targeted (SCoT) Markers

Genomic DNA was isolated from the young and healthy rice leaves during the maturity stage following a pre-standardized protocol [33]. The DNA quality was checked using 0.8% agarose gel electrophoresis and nanodrop reading [34]. The high-quality DNA was used for a polymerase chain reaction (PCR)-based genotyping using a total of 30 Start Codon Targeted (SCoT) markers, the PCR reaction was carried out in a thermal cycler (M. J. Research, MC 013130), as performed by [35], to check the polymorphic ability, and the monomorphic SCoT markers from the analysis were discarded. The list of 25 polymorphic SCoT primers used in the present investigation is presented in Table S2. The PCR-amplified products were resolved in 1.5% agarose gel following the protocol given by [36], stained using ethidium bromide (EtBr), and documented in a UV gel documentation system (Perkin Elmer, Geliance 200 imaging system, Waltham, MA, USA). The length of the amplified DNA bands was determined with reference to the 100 bp DNA ladder (Fermentas Life Sci., Waltham, MA, USA) included in the gel as a size marker. The molecular weight (nucleotide base pairs) of the most intensely amplified bands for each microsatellite marker was analyzed using AlphaEaseFC software v4.0 (Genetic Technologies, Miami, FL, USA) as reported by [14]. The electrophoretic banding pattern generated from the SCoT primers was used to calculate the pair-wise genetic similarity of rice genotypes, and a dendrogram was constructed by using unweighted pair group analysis (UPGMA) using NTSYS-pc software (version 2.02e).

### 2.7. Statistical Analyses

Statistical analysis was performed using JMP v16.0 (SAS, Cary, NC, USA) statistical software and R-programming for Windows (×64). Differences between the treatments were analyzed using Tukey–Kramer post hoc test. Analysis of variance (ANOVA) and genotype–treatment–year interaction (GxTxY) was performed using a mixed model with blocks that have random effects. Correlations between the parameters of the control set were evaluated using the corrplot package of R v4.0.3. The principal component analysis (PCA) was graphically computed using the first two—PC1 and PC2, through multivariate analysis.

## 3. Results

A total of 75 rice landraces collected from different places of Nagaland (NEI region) were initially screened for high-temperature (heat) stress tolerance based on gradual heat-responsive phenotypic changes of the leaves, i.e., days to stay healthy, leaf folding and rolling, leaf tip and margin drying, whole leaf drying, and plant death (Table S1). After the initial screening, seven landraces (Tatza (G1), Kohima special (G2), Kaladhan (G3), Laldhan (G4), Tzumma (G5), Lisem (G6), and Mapok Temeseng (G7)) were found to be high-temperature tolerant and were selected for comparison with the national check variety—N22—for further study (Table 1) to describe the interactive effects of the control (T1—no stress imposed) and the two different stress level—mild (T2) and severe (T3) with high temperatures and eCO_2_. In both the control and two stress conditions, phenological (DtoF, DtoPM), morphological (PH, LN, RL, RV, RB, SB, AnL, and P/A), physiological (CT, SPAD, NDVI, RLWC, and LA), and yield-associated traits (GY, HI, TGW, G/P, SP/P, and panicle length (PL, SF%)) were evaluated in two years/seasons (2019 and 2020).

### 3.1. Effect of High Temperatures and eCO_2_ Level on Rice Phenology, Morpho-Physiological, and Yield-Associated Parameters

The diversity in the phenological, morphological, physiological, and yield-associated traits have been detected in control and stress treatments across the two years (2019 and 2020) of study on the selected eight rice genotypes. The mean values are shown in Table 2. DtoF, RB m^−2^, TL m^−2^, and PL have been found to have the least effect in the different treatments. When comparing similar treatments in both studied years for each trait, variable performance is almost stable without much deviation. A clear trend was observed, which indicated that the mild-stress treatment (T2) yielded better variable values than the control across two years, which drastically declined under the severe stress (T3) of high temperature and eCO_2_ levels. The reduction in DtoF and DtoPM in T2 for the studied genotypes is vital for the shortening of crop cycle, which may correspond to a higher yield under stress conditions. A significant reduction in DtoF and DtoPM was observed in both T2 and T3 compared to the ambient condition, regardless of the genotypes. In T2, a reduction of 3.52% and 3.12% in DtoF was observed, while T3 showed a further reduction of 4.06% and 4.48% in both seasons. Similarly, DtoPM had a reduction of 2.90% and 1.91% in T2 and 5.66% and 5.0% in T3 across the two seasons, respectively. SPAD (chlorophyll content) values decreased significantly between the control, and treatments (T2 and T3) in both years. But the NDVI shows such a similar trend only for year-1. In year-2, NDVI increased slightly in T2 compared to the control, but it was non-significant (*p* < 0.05 in Tukey–Kramer post hoc test). It was observed that the trait values in T3 were generally much lower than the values in the control, but for some traits, it was higher than the control, e.g., CT, root traits (RL, RV, and RB), and PL.

**Figure 2:**
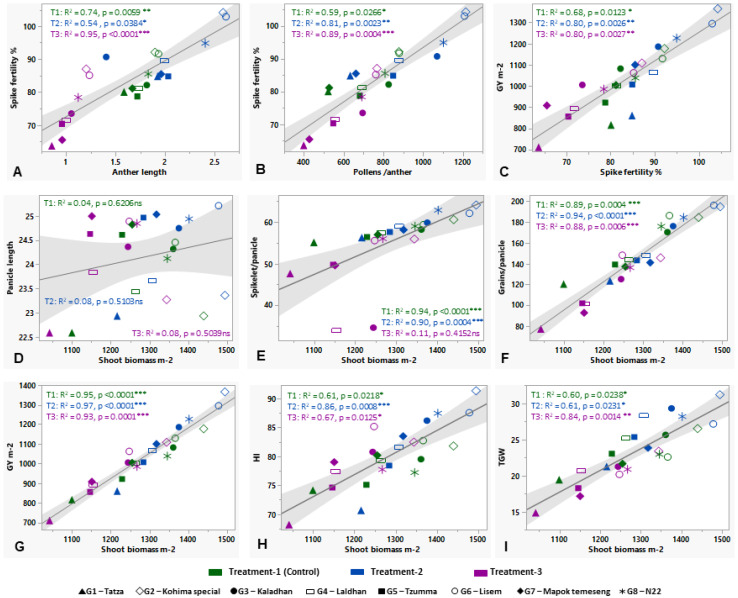
Relationship of pollen traits, biomass, and yield-associated parameters in studied rice genotypes under control and stress treatments (mild and severe). ‘*’, ‘**’, and ‘***’ denote the significance level at *p* ≤ 0.05, *p* ≤ 0.01, and *p* ≤ 0.001, respectively. ‘ns’ = Non-significant.

It is shown that anther length and pollens anther^−1^ significantly contribute to a higher SF% at maturity in the control and treatments. T2 is much more productive than the control, but severe stress (T3) dramatically reduces SF% (Figure 2A,B). In the relationship between AL and SF%, the genotypic variation in T3 is much more distinct compared to the control and T2, which is quite overlapping with P/A. Also, SF% positively and significantly influences GY (Figure 2C). A clear association of SB with major yield attributes, i.e., PL, SP/P, G/P, GY, HI, and TGW, was found (Figure 2D–I). SB is mostly positively and significantly associated with all the yield attributes, except PL, for which the association is non-significant and with a very low regression (R^2^) value (Figure 2D). SB highly influences GY, with higher significance levels and higher R^2^ values across the treatments (Figure 2G). T2 showed an increase in G/P of 3.68% and 4.03%, whereas, in contrast, T3 resulted in a stress-induced reduction of 27.10% and 26.72% in both seasonal years. The Lisem genotype exhibited a higher percent increase in G/P, with increases of 7.64% and 6.09% over the check variety N22. On average, T2 exhibited an increment in GY of 11.75% and 10.52%, and T3 showed a reduction of 8.31% and 7.87%.

**Figure 3:**
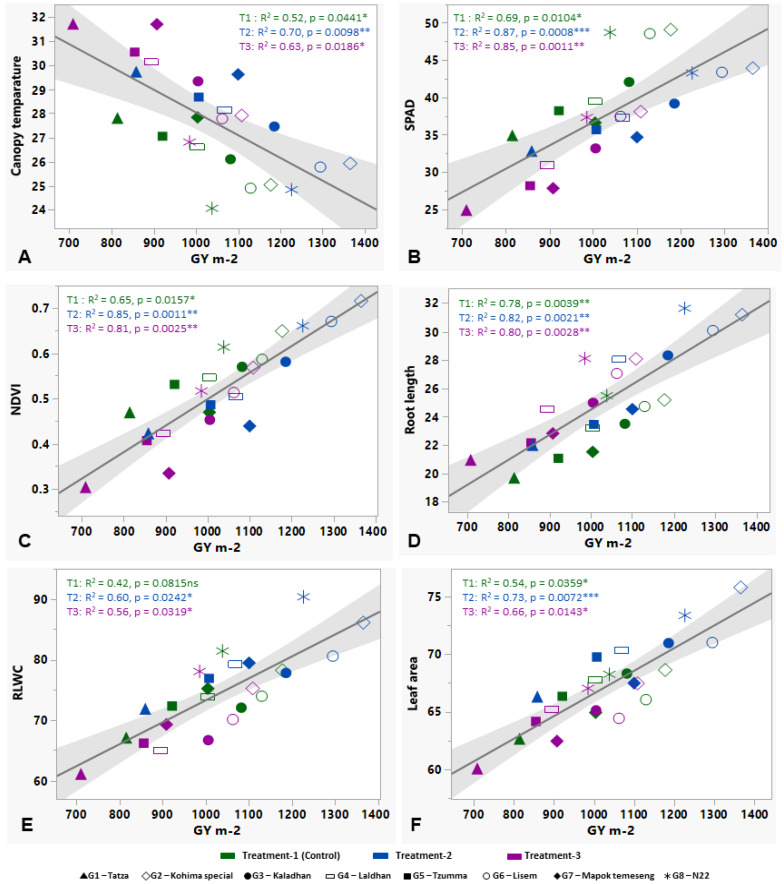
Relationship of grain yield (GY m^−2^) with the stress-responsive physiological parameters: canopy temperature (**A**), SPAD (**B**), NDVI (**C**), root length (**D**), relative leaf water content (RLWC) (**E**), and leaf area (cm^2)^ (**F**) under control and two different stress conditions (mild and severe). ‘*’, ‘**’, and ‘***’ denote the significance level at *p* ≤ 0.05, *p* ≤ 0.01, and *p* ≤ 0.001, respectively. ‘ns’ = Non-significant.

It was found that stress-responsive physiological traits, like CT, SPAD, NDVI, RL, RLWC, and LA, are significantly associated with GY m^−2^ across the control (T1) and two stress treatments—mild (T2) and severe (T3) (Figure 3A–F). CT is negatively related to the GY increment, but the rest of the physiological traits show a positive association with GY. The control and the stress treatments are significant in case of all trait-association, except when the RLWC and GY of the control has a comparatively low R^2^ value, i.e., 0.42 (Figure 3E).

### 3.2. Inter-Relationship between the Studied Traits

**Figure 4:**
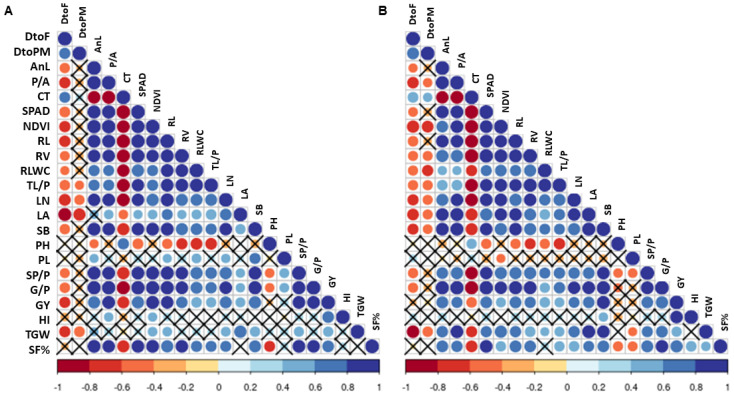
Correlation matrix in the form of corrplot using the studied parameters of rice genotypes of the control set in both year-1 (**A**) and year-2 (**B**). The range of correlation coefficient (r) is shown in the horizontal index bar. The cross mark (X) denotes a non-significant correlation (*p* = 0.05). DtoF—Days to flowering, DtoPM—days to physiological maturity, AnL—anther length, P/A—pollens per anther, CT—canopy temperature, SPAD—Soil–Plant Analysis Development, NDVI—normalized difference vegetation index, RL—root length, RV—root volume, RLWC—relative leaf water content, TL/P—tillers per plant, LN—leaf number, LA—leaf area, SB—shoot biomass, PH—plant height, PL—panicle length, SP/P—spikelets per panicle, G/P—grains per panicle, GY—grain yield, HI—harvest index, TGW—thousand-grain weight, SF%—spikelet fertility%.

The variability of the studied traits has been revealed through their inter-relationship by considering the control (T1), and the corrplot is shown in Figure 4A and Figure 4B. In both years (year-1 and year-2), DtoPM, PL, SP/P, and TGW are found to be non-significant, along with other traits. CT is shown to be mostly negatively correlated with other traits. The most positive correlation is provided by AnL, P/A, SPAD, NDVI, RL, LN, SB, SP/P, G/P, GY, and SF%. In year-1, the highest positive significant correlation (r = 0.94, *p* < 0.001) is shown between NDVI and G/P, and between P/A and GY (Figure 4A), whereas in year-2, the same highest correlation was observed for P/A, which is associated with LA and TGW (Figure 4B).

### 3.3. Combinatorial Interaction between Genotypes (G), Treatments (T) and Years (Y)

Analysis of variance (ANOVA) of the studied parameters of the eight selected rice genotypes in the two consecutive years shows enormous variability for genotypes (G), treatments (T), and years (Y), and their all combinatorial interactions (G×T, G×Y, T×Y, and G×T×Y), as shown in Table 3. ANOVA shows that the R^2^ of the traits ranged between 0.76 (PL) and 0.99 (DtoF, P/A, CT, NDVI, RB, RLWC, G/P, and SF%). Most of all traits show significant differences in the case of genotypes, treatments, and years individually, but in some instances, years do not produce significant variable differences, e.g., phenology (DtoF and DtoPM), AnL, RB, TL m^−2^, and LN. A significant difference for G×T×Y was observed in a few traits like AnL, P/A, CT, NDVI, SP/P, G/P, and SF%. In all combinatorial aspects, CT, NDVI, SF%, P/A, SPAD, LA, SP/P, and G/P were producing the maximum significant variation of the factor combinations.

### 3.4. Multivariate Analysis of the Phenotypic Parameters

**Figure 5:**
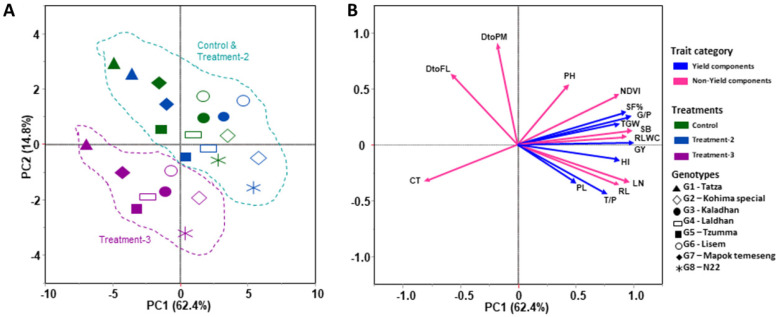
Principal component analysis (PCA) for the studied parameters of yield and non-yield components of eight rice genotypes for year-1 (season 1) under control and two treatment (treatment-2 and treatment-3) conditions. (**A**) represents the genotypic and treatment interactions, and (**B**) represents the variable vectors. Control (ambient temperature and CO_2_), treatment-2 (ambient temperature + 4 °C, CO_2_ 550 ± 20 ppm), and treatment-3 (ambient temperature + 6 °C, CO_2_ 750 ± 20 ppm). The studied traits are canopy temperature (CT), days to flowering (DtoF), days to physiological maturity (DtoPM), plant height (PH), NDVI, tiller plant^−1^ (TL/P), leaf number (LN), root length (RL), relative leaf water content (RLWC), panicle length (PL), grains panicle^−1^ (G/P), spikelets panicle^−1^ (S/P), spikelet fertility% (SF%), grain yield (GY), thousand-grain weight (TGW), and harvest index (HI).

Principal component analysis of the two consecutive years showed a clear separation of treatments with distinct trait vectors. In year-1 (Figure 5A,B), the rice genotypes were plotted with PC1, explaining 62.4% of the phenotypic variation. PC1 is positively loaded with plant traits (NDVI, SB, RLWC, LN, RL, and TL/P) and yield attributes (SF%, G/P, TGW, GY, and HI) and negatively loaded with PH and PL. Similarly, PC2 contributes 14.8% and is positively loaded with phenology (DtoF and DtoPM), and negatively loaded with CT.

**Figure 6:**
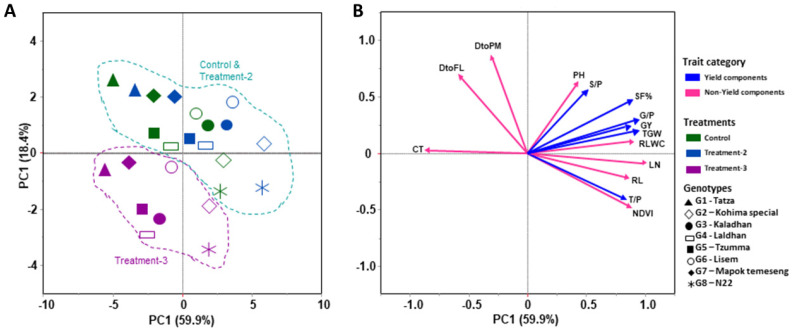
Principal component analysis (PCA) for the studied parameters of yield and non-yield components of eight rice genotypes for year-2 (season 2) under control and two treatment (treatment-2 and treatment-3) conditions. (**A**) represents the genotypic and treatment interactions, and (**B**) represents the variable vectors. Control (ambient temperature and CO_2_), treatment-2 (ambient temperature + 4 °C, CO_2_ 550 ± 20 ppm), and treatment-3 (ambient temperature + 6 °C, CO_2_ 750 ± 20 ppm). The studied traits are canopy temperature (CT), days to flowering (DtoF), days to physiological maturity (DtoPM), plant height (PH), NDVI, tiller plant^−1^ (TL/P), leaf number (LN), root length (RL), relative leaf water content (RLWC), panicle length (PL), grains panicle^−1^ (G/P), spikelets panicle^−1^ (S/P), spikelet fertility% (SF%), grain yield (GY), thousand-grain weight (TGW), and harvest index (HI).

In the second year (Figure 6A,B), almost all the vector trend and genotypic position are similar and static. PC1 and PC2 explain 59.9% and 18.4% of the trait variations, respectively. PC1 is positively loaded with plant traits (NDVI, RLWC, LN, TL/P, and RL) and yield attributes (SF%, G/P, GY, and TGW) and negatively loaded with PH and SP/P. On the other hand, PC2 contributes in the same way in year-1. Genotypic discrimination, as per the treatment, is very clear in the PCA chart, which shows that treatment-3 (severe high-temperature and eCO_2_ stress) is much more distinctive from the control and treatment-2 (mild high-temperature and eCO_2_ stress); however, that of the control and treatment-2 are almost similar and not distinctive between the genotypes.

### 3.5. Selection of Genotypes Based on Yield-Associated Performance under Stress Conditions

**Figure 7:**
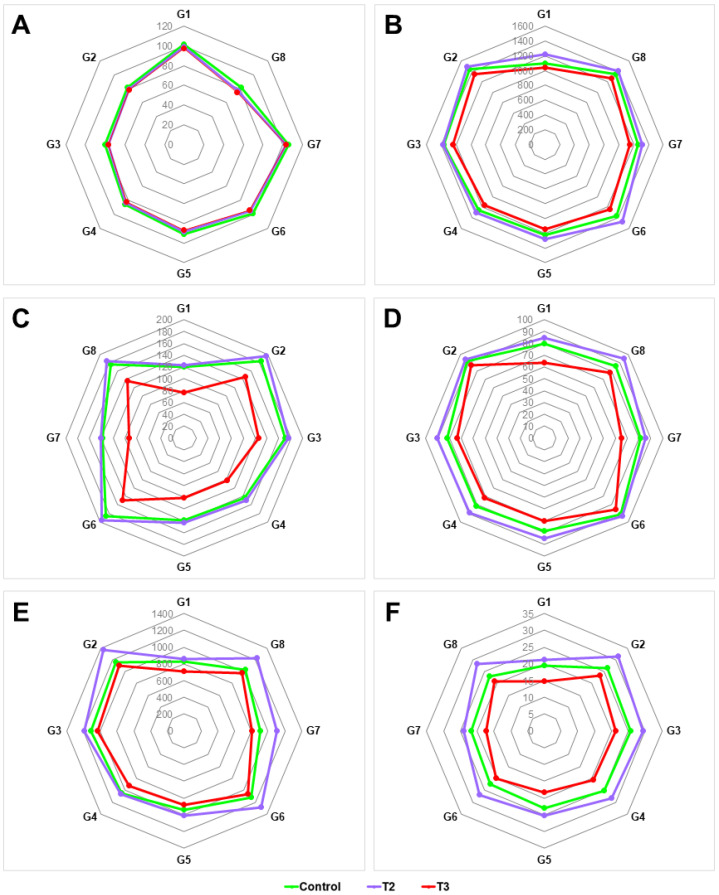
Radar charts showing the mean variability of major yield-associated traits—days to flowering (**A**), shoot biomass m^−2^ (**B**), rice-grains panicle^−1^ (**C**), spikelet fertility% (**D**), grain yield m^−2^ (**E**) and thousand-grain weight (**F**)—of studied rice genotypes across two years for the genotypic selection under different high temperature and eCO_2_ level. Genotypes are Tatza (G1), Kohima special (G2), Kaladhan (G3), Laldhan (G4), Tzumma (G5), Lisem (G6), Mapok Temeseng (G7), and N22 (G8).

Genotypic selection was based on better performance to produce improved yield and yield-associated parameters with a short phenological span under stress conditions that are comparatively higher than the check variety—N22 (Figure 7). 

Based on the fewer days needed for flowering and higher yield parameters, like SB, rice-G/P, SF%, GY, and TGW, Kohima special (G2) and Lisem (G6) were found to have a higher yield than N22 (G8). Among the genotypes, Mapok Temeseng (G7) exhibited a longer duration of flowering, with an increase of 33.11% compared to N22. On the other hand, Kaladhan had the shortest duration of flowering (19.94% less compared to N22) (Figure 7A). In the case of SF%, Kohima special exhibited the highest increase, by 9.48%, than the check genotype N22, followed by Lisem, which showed a considerable increase in SF (8.09%) (Figure 7D). Also, under the stress treatments in both T2 and T3, the performance of these two landraces is much higher compared to the other landraces. Even in the higher stress condition (T3), trait variables are decreased considerably less compared to those of the control and mild-stress treatment (T2). Out of the eight studied rice genotypes, Tatza (G1) was found to be a very low-yielding landrace under stress conditions. In the control, Kohima special and Lisem yielded 1177.50 ± 17.61 and 1129.67 ± 23.62 g rice grains m^−2^, respectively, which was much higher in range than N22 (1038.50 ± 13.43) and Tatza (814.67 ± 20.48), with the value increasing under mild stress (T2), viz. 1365.50 ± 18.44 for Kohima special, 1294.83 ± 21.73 for Lisem, 1226.67 ± 25.82 for N22, and 859 ± 29.53 for Tatza. But under severe stress (T3), Kohima special (1108.67 ± 18.61) and Lisem (1062.50 ± 12.74) performed better than N22 (985.33 ± 24.72) and Tatza (709.50 ± 34.69) (Figure 7E). Upon analyzing the GY data, a significant difference was observed among the different treatment levels. Kohima special displayed a higher per cent increase in GY (12.32%) than N22. Lisem also showed notable increases (7.30%) (Figure 7E). When considering all the treatments and comparing them to the check genotype N22, the landrace Kohima special exhibited the highest increase in TGW (by 11.22%) (Figure 7F).

### 3.6. SCoT Marker-Assisted Genetic Analysis and Genotypic Selection

Initially in this study, 30 SCoT markers were screened for genetic analysis among the studied rice genotypes, including seven landraces and one check variety of the 30 SCoTs, only 25 primers were selected for genetic analysis based on sharp, clear banding patterns. The genotyping information is presented in Table 4. The selected 25 SCoT primers amplified a total of 77 alleles, with an average of 3.08 alleles per SCoT locus. One (SCoT28 and SCoT34) to seven (SCoT21) alleles were amplified using the studied marker set that had a 100–1400 bp range for the amplicon size. Out of 77 amplified alleles, 55 alleles were found to be polymorphic with 71.42% polymorphism. Only eight markers (SCoT5, SCoT9, SCoT15, SCoT19, SCoT20, SCoT21, SCoT31, and SCoT32) were found to be only polymorphic, whereas three markers (SCoT28, SCoT34, and SCoT36) were found to be only monomorphic in nature. The estimates of polymorphic information content (PIC) values ranged from 0.22 to 0.67, with an average of 0.45 per primer. The highest PIC value (0.67) was observed for four alleles in SCoT15. The SCoT5, SCoT8, SCoT15, SCoT18, SCoT20, SCoT22, SCoT28, SCoT53, SCoT33, and SCoT35 markers showed a PIC value above 0.50. The lowest PIC value (0.22) was obtained with four alleles in the SCoT27 primer (Table 4).

**Figure 8:**
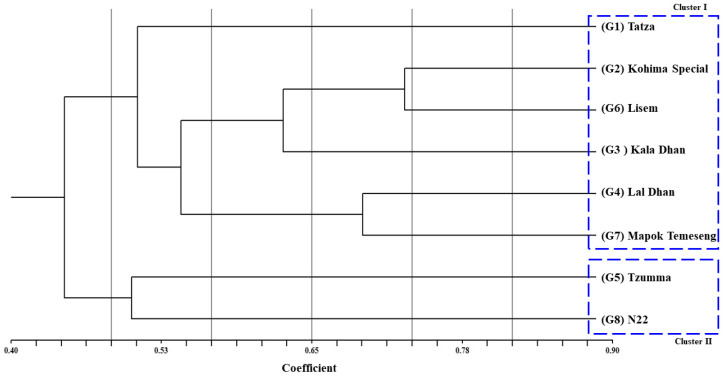
Dendrogram showing the relationship between studied rice genotypes using SCoT-marker-based genotyping data.

The dendrogram, based on SCoT-based genotyping data, classified the studied rice genotypes into two broad clusters—I and II (Figure 8). Cluster I comprise six landraces—Tatza (G1), Kohima special (G2), Kaladhan (G3), Laldhan (G4), Lisem (G6), and Mapok Temeseng (G7)—whereas cluster II comprises two genotypes—one landrace, i.e., Tzumma (G5) and one modern check variety, i.e., N22 (G8). In cluster I, Jaccard’s similarity coefficient shows the highest similarity (75%) between Kohima special and Lisem, which are connected closely to each other. Then, they sub-clustered with Kaladhan, which further sub-clustered with Laldhan and Mapok Temeseng. These five genotypes then distantly clustered with Tatza.

## 4. Discussion

The present study sheds light on the potentiality of rice landraces of the North-East Indian region to perform better than the commonly used check variety under high temperatures and eCO_2_ levels in the context of ongoing climate change in the present day. The observed variability in terms of phenological, morphological, physiological, and yield-associated traits in the studied genotypes under mild and severe treatments of high temperature and eCO_2_ in two successive cropping seasonal years (2019 and 2020) were further confirmed with SCoT-marker-based genetic profiling.

### 4.1. Rice Landraces as Pre-Breeding Materials

Rice landraces, especially from the North-East Indian (NEI) location are important pre-breeding materials for future rice-breeding programs. Numerous studies have demonstrated that indigenous rice varieties that farmers cultivated, maintained, and preserved throughout the year in their local agro-ecological niche have a high level of genetic diversity, making them viable genetic resources for enhancing yield, agronomic performance, and tolerance to abiotic stresses [16,37]. Evolutionarily, landraces play a bridging role in domestication from wild species to modern high-yielding rice varieties. In this investigation, the initial screening led to the identification of seven comparatively heat-tolerant rice landraces, out of a group of 75, that are very easy to grow and perform well during a large scale trial under field conditions. Such screening was based on the visible changes caused by the high temperature—like leaf and leaf-tip rolling, curling, and rice-plant death (Table S1) [4]. For further analysis, a national check rice variety Nagina22 (or N22) developed by the National Rice Research Institute (NRRI, Cuttack, India) was added. Many other studies used this heat-tolerant variety for a comparative study of the heat-stress response of other rice genotypes [38,39].

### 4.2. Genotypic Evaluation for Phenological, Morphological, Physiological, and Yield-Associated Traits and Traits’ Association

The evaluation of the genotypes under stress treatments reveals the profound effects of high temperatures and eCO_2_ levels on rice phenology, morpho-physiological, and yield-associated parameters (Table 2). The year-wise variability of almost every trait was very low because the traits were mostly genetically stable through the year-wise environmental fluctuations. So, further deviation must come from the treatments and genotypes. A noticeable pattern was seen wherein the mild-stress treatment (T2) of high temperature (ambient + 4 °C) and 550 ppm CO_2_ resulted in higher trait values compared to the control. However, this trend significantly diminished under much higher temperatures (ambient + 6 °C) and higher CO_2_ (750 ppm) levels (T3). The stress-induced reduction in growth parameters was greatly ameliorated by eCO_2_. Elevated CO_2_ with a high temperature shows a significant difference in plant height among the genotypes in both years. The reduction in DtoF and DtoPM under mild stress is of great importance in shortening the life cycle, which may result in higher yields under stressful conditions for the tolerant genotypes [40]. The reduction in DtoF and the increase in yield-associated traits in rice in response to high-temperature and eCO_2_ conditions, respectively, have been reported in other studies [41]. Early flowering and maturity are important to minimize the cost-effective agricultural input during the cropping season and may help to avoid the onset of climatic instabilities [42]. The treatment effect was visualized in most of the yield-associated traits as they are multigenic and very hard to regulate. For reproductive success, pollen (male reproductive particle) viability, P/A, and AnL are crucial for higher yield [33]. A significant difference in such pollen traits were reported in our previous study on hot chilis grown under high temperatures and eCO_2_ [32]. The effect of high temperature on pollen viability in rice grown under polyhouse with elevated temperature from 29 °C to 40 °C and maintained until 15 h, with a relative humidity of 75%, was observed. Such experimental set-up exhibited a 20–40% increase in SF after exposure to high temperature. Moreover, pollen fertility ranged from 39 to 90% [43]. When validating the contribution of AnL and P/A to SF% at maturity, both found a significantly positive correlation in different stress levels (Figure 2A,B), but the strength was much higher between P/A and SF% because the pollen in the anther has a direct influence on the reproductive success of rice, which in turn may eventually lead to a higher grain set panicle^−1^ and higher GY (Figure 2C). Following our results, Sakai et al. [44] also found that eCO_2_ increases the number of SP/P and grain set. An earlier study reported a higher TL number, LA, and SB (above ground) in hybrid rice cultivars under eCO_2_ conditions. The higher biomass can be also related to a higher N_2_ supply and a minimum (1–2 °C) temperature elevation [45]. The roots are also an important stress-responsive plant part as they uptakes nutrients from the soil and channel them to the shoot and leaves to influence plant growth and development. The photo-assimilates are stored in shoot and root parts, improving their biomass. Under abiotic stress conditions, there is a considerable effect on rice roots, especially root length, number, branching, and volume [14,46]. There was a notable difference in root length among the genotypes in both years. Jin et al. [47] reported that improved root traits under eCO_2_ were due to increased photosynthetic carbon allocation towards roots, which stimulates root growth and thereby enhances water and nutrients uptake efficiency. As a consequence, the RB and root/ shoot ratio in rice increased in response to higher temperatures [48]. In many cereal crops, including rice, it has been tested and proved that having a higher biomass can increase GY and grain set through channeling nutrients and photo-assimilates higher up to the growing panicles during grain set [49]. In the present study, SB significantly and positively correlates with GY, SP/P, G/P, HI, and TGW in stress treatments (Figure 2D–I) except PL (non-significant associations), which is showing a considerably lower R^2^ value, i.e., 0.76 (Table 3). Generally, it seems that a long panicle can hold higher grains, leading to a higher yield, but the finding indicates higher advantages of panicle density or grain set panicle^−1^ on the yield rather compared to PL. The probable cause may be in the mild-stress conditions, as CO_2_ helps to accelerate the photosynthesis rate due to a higher LN or greater LA, which assimilates more carbon to improve biomass, eventually boosting GY [48]. We have found the evidence that a higher LA is associated with P/A and GY in this study (Figure 4B).

### 4.3. Effect of High Temperatures and eCO_2_ Levels on Genotypes and Traits

In the previous study on hot chillis [32], three different elevated CO_2_ levels—380, 550, and 750 ppm—were used, out of which the higher concentrations show significant results to carry forward in this present experimental setup. With rice, in the present study, the mild concentration of CO_2_ (550 ppm) is higher than current atmospheric CO_2_ levels and represents a realistic projection of its concentrations in the coming years. The 4 °C increase in temperature is moderate, but it is a plausible estimation based on climate change projections. The severely elevated level (750 ppm) represents a more extreme scenario, which reflects a potential trajectory of emissions if continuing at the current rates. The 6 °C temperature increase is a severe but plausible projection under continued global warming scenarios. The IPCC provides comprehensive assessments of climate change, including future climate scenarios, CO_2_ projections, and temperature increases based on different environmental emission pathways. These predictions are utilized to select realistic stress conditions for the experiment [50]. Under high-temperature and eCO_2_ stress conditions, the physiological traits of rice play a very crucial role in characterizing genotypes and stress effects. In our study, CT, chlorophyll content (SPAD), NDVI, RL, RLWC, and LA are found to be significantly correlated with GY (Figure 3A–F). Stress-tolerant rice plants have a characteristic feature of lowering their body temperature when exposed to a stressful environment and can function internally in stress-resilient ways to produce higher grain production and/or minimize the trade-off of yield penalties because of harsh stress [51]. In this case, CT is negatively associated with the GY increase, whereas the other physiological characteristics are positively associated with GY. NDVI also indicates the vegetation and biomass of the crop and thus shows a significant correlation (r = 0.94) with G/P (Figure 4A). As was discussed earlier, the number of years (Y) has the very least effect on the traits as most of the traits show significant differences in the case of genotypes, treatments, and years individually, but in some instances where G and T are significant effects, the number of years does not produce significant variable differences for DtoF, DtoPM, AnL, RB, TL m^−2^, or LN (Table 3). Multivariate analysis exhibits a clear separation of treatments. Mainly, the severe stress condition is distinctly separated in the PCA plane due to its harsh negative effect on the studied traits, whereas the control and mild stress are almost in the same place because of their similar or close effect (Figure 5A,B). In most of the traits, mild stress has a beneficial effect over the control, as we stated before. Phenology is grouped in the close vicinity of CT. However, the plant height vector is closely situated with other physiological and yield-associated traits. In general, for the modern varieties, PH is inversely proportional to the GY as a result of the Green Revolution. But as we have used most of the landraces, it represents a higher PH-mediated biomass increment and a higher yield. When considering selecting the best-yielding rice landraces that have a similar or higher performance compared to the national check variety under stress conditions, Kohima special (G2) and Lisem (G6) have been identified as potent landraces that have a comparatively shorter DtoF and higher SB, grain set panicle^−1^, SF%, GY, and TGW (Figure 7).

### 4.4. SCoT Marker-Assisted Genetic Analysis and Genotypic Selection

In our investigation, SCoT markers were used to assess the molecular effect of high temperature and eCO_2_ on genotypes’ genetic diversity. In relation to the responsiveness of rice under other abiotic stresses, some other molecular markers, like SSR, ISSR, SNP, were used in the rice landrace population [13,14,34,36,52]. SCoT-marker-based analysis is better than other frequently used molecular markers since primer creation does not need genomic sequence information [23]. SCoT markers also have larger polymorphism rates than SSRs, making them better at distinguishing closely related genotypes. SCoT analysis eliminates the need for resource-intensive SNP identification and genotyping, making it cheaper than SNP markers. SCoT markers also target non-coding areas around start codons, which may identify gene expression and functional variation regulators [53,54,55]. In our study, eight SCoT markers (SCoT5, SCoT9, SCoT15, SCoT19, SCoT20, SCoT21, SCoT31, and SCoT32) have been identified as highly polymorphic and can be used further in rice genotyping for high-temperature- and CO_2_-induced stress. The polymorphism information content (PIC) of the studied SCoT markers is moderate and signifies each marker’s genotypic discrimination ability. The two best-performing rice landraces (Kohima special and Lisem) under high temperature and eCO_2_ level were determined by multivariate analysis, which is supported by the SCoT-marker-based selection data as they showed a similarity in their marker-based clustering (Figure 8).

In this way, rice landraces exhibiting resilience and superior performance under high-temperature and elevated atmospheric-CO_2_-stress conditions should be prioritized in ongoing climate change scenarios in agricultural sectors. However, stability over multiple environments and seasons is critical, so multi-location trials should be conducted to validate their adaptability. Additionally, assessing the genetic diversity present in the selected rice landraces will guide breeders in avoiding narrow genetic bases and potential vulnerabilities. New climate-resistant rice varieties may be developed using the genetic diversity of Kohima Special and Lisem. These landraces can provide a sustainable rice production in the face of climate change by incorporating traits like efficient photosynthesis, heat tolerance, and water-use efficiency. This will improve food security for local communities and the wider population.

## 5. Conclusions and Future Perspectives

The present investigation employed the evaluation of rice landraces for better performance under high temperatures and eCO_2_ for breeding traits, and was further aided by SCoT-marker-assisted selection. The study aimed to identify and understand the diverse responses of Nagaland’s rice genotypes to the changing environmental factors, providing critical insights into their adaptive mechanisms. The novelty of this study lies in the used rice landraces, which are very much native to the unexplored regions of North-East India (NEI), with their potential resilience to these climate change-related stressors, highlighting the importance of regional diversity and adaptation. By comprehensively assessing morphological, phenological, physiological, and yield-associated traits, this finding led to the identification of two potent regional rice landraces—Kohima special and Lisem—which have a much superior yield ability, compared to the national check variety N22. SCoT-marker-based genotyping also revealed moderate genetic diversity among the genotypes and found few potent markers (SCoT5, SCoT9, SCoT15, SCoT19, SCoT20, SCoT21, SCoT31, and SCoT32) with better discrimination ability to precisely select the superior genotypes that would help rice breeders in the selection of suitable parents for breeding purpose and genetic mapping studies. By using these two landraces as donor parents, the genetic background of N22 and other modern cultivars can be improved and sustained in changing climatic scenarios. The unique geographic focus, with emphasis on future climatic stressors; comprehensive evaluation, molecular analysis, and the identification of resilient rice landraces collectively differentiate this study from others in the field of rice research and stress-tolerance assessment. Furthermore, it is expected that the insights gained from this study will aid in the development of climate-resilient rice varieties capable of withstanding future climatic challenges. The findings could influence rice-breeding programs, guiding the selection of rice varieties that are better equipped to thrive in the changing environmental conditions. This multidimensional perspective can shape the future of rice breeding and contribute to global efforts aimed at achieving food security in an era of environmental uncertainty.

## Figures and Tables

**Table 1:** Shortlisted rice genotypes’ names, collection places from Nagaland state of India, and genotypic types.

Accession No.	Rice Genotypes	Places of Collection	District	State	Genotypic Type
G1	Tatza	Punglao	Dimapur	Nagaland	Landrace
G2	Kohima Special	Molva	Dimapur	Nagaland	Landrace
G3	Kaladhan	Jharnapani	Dimapur	Nagaland	Landrace
G4	Laldhan	Medziphema	Dimapur	Nagaland	Landrace
G5	Tzumma	Sochunoma	Dimapur	Nagaland	Landrace
G6	Lisem	Mopongchuket	Mokokchung	Nagaland	Landrace
G7	MapokTemeseng	Merangkong	Mokokchung	Nagaland	Landrace
G8	Nagina 22 (N22)	Central Rice Research Institute (CRRI), Cuttack, India	Bidyadharpur	Orissa	Improved (check variety)

**Table 2:** Mean values of the studied traits under the control and two different stress treatments in two consecutive years—year-1 and year-2. The mean values with different letters are significantly different (*p* = 0.05) as per Tukey–Kramer’s LSD test.

Traits	Year-1			Year-2			Mean (*X*)
	Control	T-2	T-3	Control	T-2	T-3	
Days to flowering	90.71 ^a^	87.57 ^a^	87.09 ^a^	90.63 ^a^	87.85 ^a^	86.61 ^a^	88.41
Days to physiological maturity	121.54 ^a^	118.02 ^ab^	114.65 ^b^	120.30 ^a^	118.01 ^ab^	114.32 ^b^	117.81
Anther length (mm)	1.79 ^bc^	2.06 ^ab^	1.01 ^d^	1.76 ^c^	2.17 ^a^	1.09 ^d^	1.65
Pollens anther^−1^	715.78 ^b^	939.73 ^a^	596.77 ^c^	737.08 ^b^	976.06 ^a^	613.94 ^c^	763.23
Canopy temperature (°C)	26.45 ^c^	27.90 ^b^	30.45 ^a^	25.91 ^c^	27.15 ^bc^	28.56 ^b^	27.74
SPAD	40.91 ^ab^	37.82 ^b^	31.50 ^c^	43.51 ^a^	39.75 ^ab^	33.01 ^c^	37.75
NDVI	0.69 ^a^	0.66 ^a^	0.45 ^b^	0.42 ^b^	0.46 ^b^	0.43 ^b^	0.52
Root length (mm)	22.81 ^b^	27.51 ^a^	24.57 ^b^	23.27 ^b^	27.31 ^a^	25.10 ^ab^	25.09
Root volume	43.87 ^c^	48.89 ^ab^	46.22 ^bc^	44.48 ^c^	49.28 ^a^	46.76 ^abc^	46.58
Root biomass m^−2^	41.28 ^a^	46.12 ^a^	43.15 ^a^	41.48 ^a^	45.83 ^a^	43.33 ^a^	43.53
RLWC	74.75 ^b^	81.14 ^a^	69.34 ^c^	73.76 ^b^	79.38 ^a^	68.55 ^c^	74.48
Tillers m^−2^	594.67 ^a^	627.08 ^a^	579.71 ^a^	594.67 ^a^	624.13 ^a^	582.08 ^a^	600.39
Leaf number	23.47 ^ab^	24.59 ^a^	22.85 ^b^	23.43 ^ab^	24.43 ^a^	22.62 ^b^	23.56
Leaf area (cm^2^)	66.30 ^bc^	69.64 ^a^	64.28 ^c^	66.92 ^b^	71.60 ^a^	64.70 ^bc^	67.24
Shoot biomass m^−2^	1311.17 ^ab^	1370.04 ^a^	1209.42 ^cd^	1278.58 ^bc^	1348.92 ^ab^	1189.13 ^d^	1284.54
PH (cm)	111.76 ^b^	118.15 ^a^	105.04 ^c^	111.80 ^b^	119.94 ^a^	104.97 ^c^	111.94
Panicle length (cm)	23.80 ^a^	24.16 ^a^	24.08 ^a^	24.02 ^a^	24.56 ^a^	24.27 ^a^	24.15
Spikelets panicle^−1^	58.67 ^ab^	61.10 ^a^	52.27 ^b^	57.17 ^ab^	58.96 ^ab^	43.50 ^c^	55.28
Grains panicle^−1^	157.55 ^a^	163.61 ^a^	116.01 ^b^	156.92 ^a^	163.46 ^a^	116.08 ^b^	145.61
GY g m^−2^	1019.29 ^bc^	1141.79 ^a^	937.21 ^c^	1023.42 ^bc^	1134.79 ^ab^	944.83 ^c^	1033.56
HI	77.68 ^b^	83 ^a^	77.18 ^b^	79.86 ^ab^	83.72 ^a^	79.19 ^ab^	80.11
TGW	24.15 ^bc^	27.47 ^a^	20.14 ^d^	22.64 ^c^	26.23 ^ab^	19.07 ^d^	23.28
Spike fertility%	83.40 ^b^	91.23 ^a^	74.16 ^c^	84.81 ^b^	93.20 ^a^	74.71 ^c^	83.58

**Table 3:** Analysis of variance (ANOVA) of the studied parameters of rice genotypes with genotypes (G), treatments (T), and years (Y), and their interactions.

Fixed Factors	DF	Studied Parameters					
		DtoF	DtoPM	AnL	P/A	CT	SPAD
G	7	<0.0001 ***	<0.0001 ***	<0.0001 ***	<0.0001 ***	<0.0001 ***	<0.0001 ***
T	2	<0.0001 ***	<0.0001 ***	<0.0001 ***	<0.0001 ***	<0.0001 ***	<0.0001 ***
Y	1	0.7054	0.035 *	0.1941	<0.0001 ***	<0.0001 ***	<0.0001 ***
G×T	14	0.1518	0.0888	<0.0001 ***	<0.0001 ***	<0.0001 ***	<0.0001 ***
G×Y	7	0.0544	<0.0001 ***	0.0139 *	<0.0001 ***	<0.0001 ***	<0.0001 ***
T×Y	2	0.4244	0.1119	0.3264	0.1361	<0.0001 ***	0.0312 *
G×T×Y	14	0.9984	0.9711	0.0057**	<0.0001 ***	<0.0001 ***	0.9573
*R* ^2^		*0.99*	*0.96*	*0.89*	*0.99*	*0.99*	*0.98*
**Fixed factors**	**DF**	**NDVI**	**RL**	**RV**	**RB**	**RLWC**	**TL/P**
G	7	<0.0001 ***	<0.0001 ***	<0.0001 ***	<0.0001 ***	<0.0001 ***	<0.0001 ***
T	2	<0.0001 ***	<0.0001 ***	<0.0001 ***	<0.0001 ***	<0.0001 ***	<0.0001 ***
Y	1	<0.0001 ***	<0.0001 ***	<0.0001 ***	0.732	<0.0001 ***	0.9692
G×T	14	<0.0001 ***	<0.0001 ***	<0.0001 ***	<0.0001 ***	<0.0001 ***	0.0372 *
G×Y	7	<0.0001 ***	0.0057 **	0.0313 *	0.8495	<0.0001 ***	0.0394 *
T×Y	2	<0.0001 ***	0.0418 *	0.7323	0.0812	0.0038 **	0.8749
G×T×Y	14	<0.0001 ***	0.99	0.996	0.994	0.4069	0.9938
*R* ^2^		*0.99*	*0.97*	*0.98*	*0.99*	*0.99*	*0.97*
**Fixed factors**	**DF**	**LN**	**LA**	**LAI**	**SB**	**PH**	**PL**
G	7	<0.0001	<0.0001 ***	<0.0001 ***	<0.0001 ***	<0.0001 ***	<0.0001 ***
T	2	<0.0001	<0.0001 ***	<0.0001 ***	<0.0001 ***	<0.0001 ***	0.0087 *
Y	1	0.1831	<0.0001 ***	<0.0001 ***	<0.0001 ***	0.0067 **	0.0218 *
G×T	14	0.5779	<0.0001 ***	<0.0001 ***	<0.0001 ***	<0.0001 ***	0.9957
G×Y	7	0.0099 **	<0.0001 ***	<0.0001 ***	0.9057	0.1162	<0.0001 ***
T×Y	2	0.7737	<0.0001 ***	0.6975	0.2438	0.0006 ***	0.6283
G×T×Y	14	0.9998	0.7161	0.7323	0.9393	0.9945	0.9403
*R* ^2^		*0.92*	*0.96*	*0.98*	*0.98*	*0.97*	*0.76*
**Fixed factors**	**DF**	**SP/P**	**G/P**	**GY**	**HI**	**TGW**	**SF%**
G	7	<0.0001	<0.0001 ***	<0.0001 ***	<0.0001 ***	<0.0001 ***	<0.0001 ***
T	2	<0.0001	<0.0001 ***	<0.0001 ***	<0.0001 ***	<0.0001 ***	<0.0001 ***
Y	1	<0.0001	<0.0001 ***	<0.0001 ***	<0.0001 ***	<0.0001 ***	<0.0001 ***
G×T	14	<0.0001	<0.0001 ***	<0.0001 ***	<0.0001 ***	<0.0001 ***	<0.0001 ***
G×Y	7	0.0012 **	<0.0001 ***	<0.0001 ***	<0.0001 ***	<0.0001 ***	<0.0001 ***
T×Y	2	0.0064 **	0.2735	0.3247	0.2719	0.1851	0.0035 **
G×T×Y	14	<0.0001 ***	<0.0001 ***	0.8294	0.6204	0.2886	0.0418 *
*R* ^2^		*0.77*	*0.99*	*0.98*	*0.90*	*0.98*	*0.99*

G—genotype, T—treatments, Y—years, DF—degree of freedom, DtoF—days to flowering, DtoPM—days to physiological maturity, AnL—anther length, P/A—pollens per anther, CT—canopy temperature, SPAD, NDVI—normalized difference vegetation index, RL—root length, RV—root volume, RB—root biomass, RLWC—relative leaf water content, TL/P—tillers per plant, LN—leaf number, LA—leaf area, LAI—leaf area index, SB—shoot biomass, PH—plant height, PL—panicle length, SP/P—spikelets per panicle, G/P—grains per panicle, GY—grain yield, HI—harvest index, TGW—thousand-grain weight, SF%—spikelet fertility%. ‘*’, ‘**’, and ‘***’ denote the significance level at *p* ≤ 0.05, *p* ≤ 0.01, and *p* ≤ 0.001, respectively.

**Table 4:** Molecular genotyping data through the 25 SCoT-marker-based fingerprinting of rice genotypes, including landraces and check variety.

Sl. No.	SCoT Markers	Total Amplicons	Amplicon Size (bp)	Number of Monomorphic Bands (%)	Number of Polymorphic Bands (%)	PIC ^1^
1	SCoT2	2	180–300	1 (50%)	1 (50%)	0.44
2	SCoT5	3	100–400	0	3 (100%)	0.52
3	SCoT6	6	150–750	1 (16.66%)	5 (83.33%)	0.38
4	SCoT8	4	300–800	2 (50%)	2 (50%)	0.58
5	SCoT9	2	140–190	0	2 (100%)	0.30
6	SCoT15	4	190–1050	0	4 (100%)	0.67
7	SCoT16	3	160–480	1 (33.33%)	2 (66.66%)	0.50
8	SCoT17	4	110–820	1 (25%)	3 (75%)	0.43
9	SCoT18	3	900–1400	1 (33.33%)	2 (66.66%)	0.60
10	SCoT19	2	120–260	0	2 (100%)	0.49
11	SCoT20	2	170–350	0	2 (100%)	0.55
12	SCoT21	7	220–900	0	7 (100%)	0.37
13	SCoT22	4	400–1000	1 (25%)	3 (75%)	0.58
14	SCoT24	3	400–1200	2 (66.66%)	1 (33.33%)	0.34
15	SCoT26	3	400–1250	2 (66.66%)	1 (33.33%)	0.38
16	SCoT27	4	200–550	1 (25%)	3 (75%)	0.22
17	SCoT28	1	1000	1 (100%)	0	0.52
18	SCoT29	4	300–1200	2 (50%)	2 (50%)	0.47
19	SCoT30	4	200–800	1 (25%)	3 (75%)	0.53
20	SCoT31	2	300–500	0	2 (100%)	0.29
21	SCoT32	2	120–280	0	2 (100%)	0.38
22	SCoT33	3	150–500	1 (33.33%)	2 (66.66%)	0.58
23	SCoT34	1	800	1 (100%)	0	0.30
24	SCoT35	2	180–500	1 (50%)	1 (50%)	0.57
25	SCoT36	2	300–600	2 (100%)	0	0.33
	Total	77		22	55	
	Average	3.08		0.88	2.2	0.45

^1^ Polymorphism information content (PIC).

## Data Availability

The data presented in this study are available on request.

## Supplementary Materials

The following supporting information can be downloaded at: https://www.mdpi.com/article/10.3390/plants12203655/s1, Figure S1: Meteorological data of maximum and minimum temperature (°C), total rainfall (mm), and relative humidity (RH%) in the morning and evening during the cropping seasons (A) Year-1 and (B) Year-2; Table S1: Screening of rice landraces from North East India (Nagaland) under high temperature stress condition; Table S2: Details of used SCoT primers and their sequences, GC content and melting temperature (Tm).
