# Supplementary material for: Evaluation of Morpho-Physiological and Yield-Associated Traits of Rice (Oryza sativa L.) Landraces Combined with Marker-Assisted Selection under High-Temperature Stress and Elevated Atmospheric CO2 Levels
Source: Plants (Basel). 2023 Oct 23;12(20):3655. doi: 10.3390/plants12203655 (PMC10610436; doi:10.3390/plants12203655)
Supplement: Supplementary file 1 [file plants-12-03655-s001.zip › plants-2636476-supplementary.pdf]

Supplementary file

A

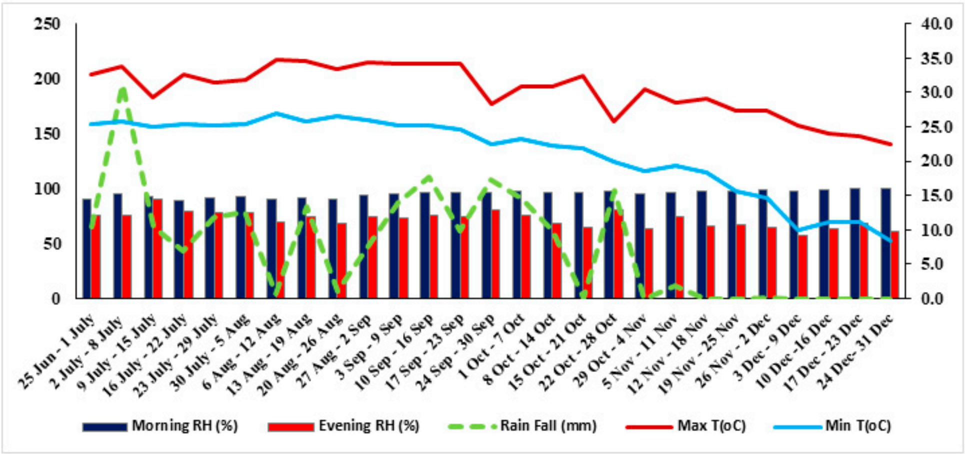

B

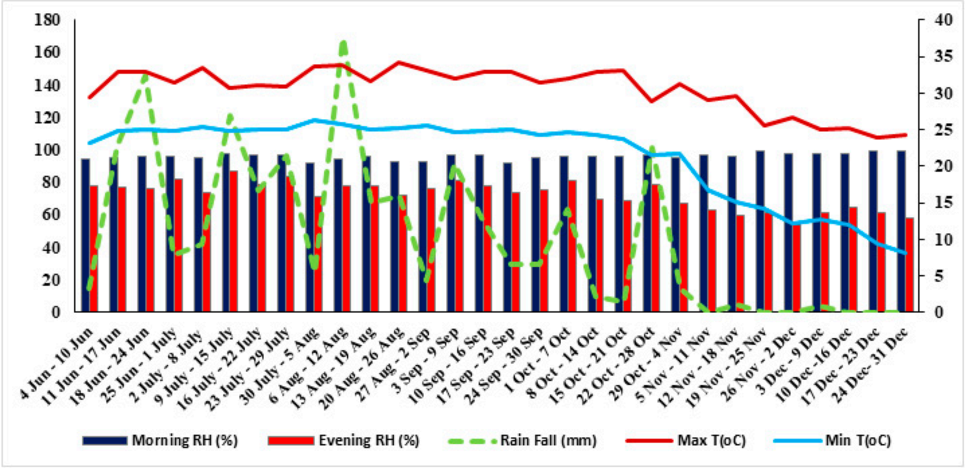

Figure S1. Meteorological data of maximum and minimum temperature (°C), total rainfall (mm), and relative humidity (RH%) in the morning and evening during the cropping seasons (A) Year-1 and (B) Year-2.

Table S1. Screening of rice landraces from North East India (Nagaland) under high temperature stress condition.

Rice landraces	Collection place of Nagaland	Days of healthiness	Days to leaf fold	Days to leaf rolling in to V shaped	Days to leaf margin (o shaped)	Days to leaf rolling (U shaped)	Days to leaf fully rolled	Days to slight tip drying	Days to tip drying extend to ¼	Days to ¼ to 1/5 of leaf fully dried	Days to more than 2/3 rd of leaf fully dried	Days to plant die	Remarks
SARS-10	Yisemyong	2	3	4	6	7	9	18	20	21	22	23	ST
Tsukmerem	Yisemyong	3	5	7	9	10	12	18	20	22	24	25	MT
SARS-3	Yisemyong	4	7	9	11	12	14	18	17	20	21	22	ST
Masutsuk	Yisemyong	3	5	7	9	11	13	18	20	22	24	25	MT
Nailongmapok	Yisemyong	2	3	4	6	7	9	18	20	22	23	24	ST
Longsatsuk	Yisemyong	2	3	4	6	7	9	18	20	21	22	23	ST
Tangmatsuk	Yisemyong	2	3	4	5	7	8	15	18	20	21	22	ST
Koyatsuk	Yisemyong	2	3	4	6	7	9	18	20	21	22	23	ST
Chakpamapok	Yisemyong	3	5	7	9	10	12	18	20	22	24	25	MT
Manentsuk	Yisemyong	2	4	6	9	11	14	20	22	24	26	28	MT
Tsuknak	Yisemyong	5	7	8	13	15	17	18	20	22	24	30	HT
Tsulotsuk	Yisemyong	2	4	6	9	11	14	20	22	24	26	28	MT
Lisemtsuk	Mepongchuket	2	4	6	9	11	14	20	22	24	28	34	HT
Phomtsuk	Yisemyong	5	7	8	13	15	17	18	20	22	24	28	MT
Mopoklongjang	Mepongchucket	2	4	6	9	11	14	20	22	24	26	28	MT
Longkongtsuk	Mepongchuket	2	4	6	9	11	14	20	22	24	26	28	MT
SARS-7	Yisemyong	5	7	8	13	15	17	18	20	22	24	30	HT
Teke	Yisemyong	2	4	6	9	11	14	20	22	24	26	28	MT
SARS-8	Yisemyong	2	4	6	9	11	14	20	22	24	26	28	MT
SARS-9	Yisemyong	5	7	8	13	15	17	18	20	22	24	31	HT
Lisemtsuk 1	Mepongchuket	2	4	6	9	11	14	20	22	24	26	28	MT
SARS-11	Yisemyong	2	4	6	9	11	14	20	22	24	26	28	MT
SARS-6	Yisemyong	5	7	8	13	15	17	18	20	22	24	32	HT
Yam	Mon	2	4	6	9	11	14	20	22	24	26	28	MT
Chapanga	Mon	2	4	6	9	11	14	20	22	24	26	28	MT
Chakad	Mon	5	7	8	13	15	17	18	20	22	24	32	HT
Gotzang	Mon	2	4	6	9	11	14	20	22	24	26	28	MT
Kaladhan	Mon	2	4	6	9	11	14	20	22	24	26	28	MT
Khadhu	Mon	5	7	8	13	15	17	18	20	22	24	28	HT
Wam	Mon	2	4	6	9	11	14	20	22	24	26	28	MT
Mapokmerangkong	Merengkong	2	4	6	9	11	14	20	22	24	26	28	MT
Temeremmapok	Mupongchucket	2	4	6	9	11	14	20	22	24	26	28	MT
Tsukmesung	Mupongchucket	5	7	8	13	15	17	18	20	22	24	32	HT
Temeremtsuk	Mupongchucket	2	4	6	9	11	14	20	22	24	26	28	MT
Temesungtsuk	Mupongchucket	2	4	6	9	11	14	20	22	24	26	28	MT
Ahari	Jharnapani	5	7	8	13	15	17	18	20	22	24	31	HT
Sceiti	Jharnapani	2	4	6	9	11	14	20	22	24	26	29	MT
Nldspl 1	Jharnapani	2	4	6	9	11	14	20	22	24	26	30	MT
Kohima special	Jharnapani	5	7	8	13	15	17	18	20	24	26	35	HT
Baiti	Punglao	4	6	9	11	13	15	20	22	24	26	28	MT
Tatza	Punglao	7	12	15	18	21	24	28	29	30	32	34	HT
Gluthpunglaw	Punglao	2	3	4	6	7	9	12	13	15	17	19	ST
Joha	Medziphema	3	5	7	9	10	11	18	20	22	24	25	MT
Tzumma	Medziphema	4	6	9	11	13	15	20	22	26	28	33	HT

Manipurspl	Tizit	2	4	6	8	10	12	16	18	20	21	24	MT
Kamkum	Tizit	4	6	9	11	13	15	20	22	24	26	28	MT
Burdhan	Tizit	7	12	15	18	18	22	26	27	27	28	31	MT
Hatau	Sangsa	2	3	4	6	7	9	12	13	15	17	19	ST
Phusa	Aboi	3	5	7	9	10	11	18	20	22	24	25	MT
Thanyu	Mon	4	6	9	11	13	15	20	22	24	26	28	MT
Yamshang	Aboi	2	4	6	8	10	12	16	18	20	21	24	ST
Phula	Aboi	4	6	9	11	13	15	20	22	24	26	28	MT
Wangsho	Aboi	7	12	15	18	21	24	25	26	28	2 9	31	HT
Manen 1	Mongchen	2	3	4	6	7	9	12	13	15	17	19	ST
Manen 2	Mongchen	3	5	7	9	10	11	18	20	22	24	25	MT
Manen 3	Mongchen	4	6	9	11	13	15	20	22	23	25	26	MT
Lisemtsuk 1	Mongchen	2	4	6	8	10	12	16	20	24	28	31	HT
Nld spl2	Medziphema	4	6	9	11	13	15	20	22	26	28	31	HT
Kaladhan	Jharnapani	7	12	15	18	21	24	28	29	30	32	35	HT
Nldspl	Jharnapani	7	12	15	18	21	24	25	26	28	2 9	31	HT
Masu 1	Mongchen	3	5	7	9	10	11	18	20	22	24	25	MT
Masu 2	Mongchen	4	6	9	11	13	15	20	22	24	26	28	MT
Masu 3	Mongchen	2	4	6	8	10	12	16	18	20	21	24	ST
Lisemtsuk 2	Mongchen	7	12	15	18	21	24	28	29	30	32	33	HT
Mapoktemeseng	Merangkong	7	12	15	18	21	24	28	29	30	32	35	HT
Masutsuk 1	Merangkong	2	3	4	6	7	9	12	13	15	17	19	ST
Tsukmerem	Merangkong	3	5	7	9	10	11	18	20	22	24	25	MT
Temesungtsuk	Merangkong	4	6	9	11	13	15	20	22	24	26	28	MT
Changtah	Mon	2	4	6	8	10	12	16	18	20	21	24	MT
Phuaha	Mon	4	6	9	11	13	15	20	22	24	26	28	MT
Humo	Molva	7	12	15	18	21	24	28	29	30	32	33	HT
Khaja	Molva	2	3	4	6	7	9	12	13	15	17	19	ST
Bermol	Molva	3	5	7	9	10	11	18	20	22	24	25	MT
Laldhan	Molva	4	6	9	11	15	20	22	24	26	28	34	HT
Mujangka	Longpayimsen	2	4	6	8	10	12	16	18	23	25	29	MT

HT - Highly tolerant = Plants were survived more than 30 days;
MT – Moderate tolerant = Plants were survived 25-30 days;
ST - Susceptible = Plants were died within 20 days;

Table S2. Details of used SCoT primers and their sequences, GC content and melting temperature (Tm).

Sl. No.	Primers	Sequences (5'-3')	GC content (%)	T_m (°C)
1.	SCoT2	CAACAATGGCTACAACCC	55.6	53.6
2.	SCoT5	CAACAATGGCTACCACGA	50.0	52.6
3.	SCoT6	CAACAATGGCTACCACGC	55.6	54.4
4.	SCoT8	CAACAATGGCTACCACGT	50.0	52.9
5.	SCoT9	CAACAATGGCTACCAGCA	50.0	52.9
6.	SCoT15	ACGACATGGCGACCGCGA	67.0	59.0
7.	SCoT16	ACCATGGCTACCACCGAC	56.0	60.7
8.	SCoT17	ACCATGGCTACCACCGAG	67.0	59.0
9.	SCoT18	ACCATGGCTACCACCGCC	67.0	59.0
10.	SCoT19	ACCATGGCTACCACCGGC	66.7	60.7
11.	SCoT20	ACCATGGCTACCACCGCG	66.7	58.3
12.	SCoT21	ACGACATGGCGACCCACA	61.0	57.0
13.	SCoT22	AACCATGGCTACCACCAC	56.0	55.0
14.	SCoT24	CACCATGGCTACCACCAT	61.2	57.0
15.	SCoT26	ACCATGGCTACCACCGTC	61.1	57.3
16.	SCoT27	ACCATGGCTACCACCGTG	61.0	57.0
17.	SCoT28	CCATGGCTACCACCGCCA	66.7	60.7
18.	SCoT29	CCATGGCTACCACCGGCC	72.0	61.5
19.	SCoT30	CCATGGCTACCACCGGCG	72.2	61.8
20.	SCoT31	CCATGGCTACCACCGCCT	66.7	60.4
21.	SCoT32	CCATGGCTACCACCGCAC	66.7	59.1
22.	SCoT33	CCATGGCTACCACCGCAG	66.7	58.3
23.	SCoT34	ACCATGGCTACCACCGCA	61.0	57.2
24.	SCoT35	CATGGCTACCACCGGCCC	72.2	61.7
25.	SCoT36	GCAACAATGGCTACCACC	55.6	54.2
